# Bridging the Gap in Post-Fracture Care Using the RE-AIM Framework: Insights From the OPTIONS Study

**DOI:** 10.1093/geroni/igaf122.4080

**Published:** 2025-12-31

**Authors:** Gretchen Tucker, Veysel Baris, Min Jeoung Kang, Nancy Latham, Kumiko Schnock, Pamela Garabedian, Denise Orwig, Patricia Dykes

**Affiliations:** University of Maryland, Baltimore School of Medicine, Baltimore, Maryland, United States; Dokuz Eylul University, Izmir, Izmir, Turkey; Brigham and Womens Hospital, Boston, Massachusetts, United States; Brigham and Women’s Hospital, Boston, Massachusetts, United States; Brigham and Women’s Hospital, Boston, Massachusetts, United States; Mass General Brigham, Inc., Somerville, Massachusetts, United States; University of Maryland, Baltimore, Baltimore, Maryland, United States; BWH/Harvard Medical School, Boston, Massachusetts, United States

## Abstract

An estimated 10 million older Americans have osteoporosis, a leading risk factor for fragility fractures that often result in disability, diminished quality of life, and increased mortality. Despite availability of effective treatments, evidence-based post-fracture care remains underutilized. The OsteoPorotic fracTure preventION System (OPTIONS) is an integrated, multi-modal intervention designed to improve care for community-dwelling older adults following lower limb fractures who transition to skilled nursing facilities and back home. OPTIONS includes clinical decision support tools for providers, patients, and care partners to strengthen fracture prevention efforts across care transitions. In the pre-implementation phase, we used the RE-AIM (Reach, Effectiveness, Adoption, Implementation Maintenance) implementation science framework to explore current fracture care practices in skilled nursing facilities (e.g., exercise, nutrition, and bone health medications) and explored facilitators and barriers to intervention delivery and uptake. We conducted seven interdisciplinary focus groups with physical and occupational therapists, nurses, and dieticians using a semi-structured interview guide. Transcripts were coded and data were analyzed using content analysis. Barriers identified were patients’ complex health conditions, staff turnover, and osteoporosis medication costs. Facilitators included strong communication among staff, access to resources, sufficient training, and training offered in multiple formats. Findings were used to inform development of a practical OPTIONS implementation toolkit, including training materials, workflow guides, and care team resources. The toolkit is currently being tested during the study’s pilot phase to assess feasibility and inform broader implementation. This work aims to improve the delivery of fracture prevention interventions and reduce secondary fracture risk among vulnerable older adults.

